# Age‐associated accumulation of RAB9 disrupts oocyte meiosis

**DOI:** 10.1111/acel.14449

**Published:** 2024-12-15

**Authors:** Min Gao, Fang Wang, Tengteng Xu, Yanling Qiu, Tianqi Cao, Simiao Liu, Wenlian Wu, Yitong Zhou, Haiying Liu, Fenghua Liu, Junjiu Huang

**Affiliations:** ^1^ Key Laboratory of Reproductive Medicine of Guangdong Province, The First Affiliated Hospital and School of Life Sciences Sun Yat‐Sen University Guangzhou China; ^2^ MOE Key Laboratory of Gene Function and Regulation, State Key Laboratory of Biocontrol, School of Life Sciences Sun Yat‐Sen University Guangzhou China; ^3^ Key Laboratory of Reproductive Health Diseases Research and Translation of Ministry of Education, The First Affiliated Hospital Hainan Medical University Haikou China; ^4^ Department of Reproductive Medical Center Guangdong Women and Children Hospital Guangzhou China; ^5^ Department of Gynecology, Clinical Transformation and Application Key Lab for Obstetrics and Gynecology, Pediatrics, and Reproductive Medicine of Jiangmen Jiangmen Central Hospital Jiangmen China

**Keywords:** aging, meiosis, mitochondrial function, oocyte, RAB9

## Abstract

The critical role of some RAB family members in oocyte meiosis has been extensively studied, but their role in oocyte aging remains poorly understood. Here, we report that the vesicle trafficking regulator, RAB9 GTPase, is essential for oocyte meiosis and aging in humans and mice. RAB9 was mainly located at the meiotic spindle periphery and cortex during oocyte meiosis. In humans and mice, we found that the RAB9 protein level were significantly increased in old oocytes. Age‐related accumulation of RAB9 inhibits first polar body extrusion and reduces the developmental potential of oocytes. Further studies showed that increased Rab9 disrupts spindle formation and chromosome alignment. In addition, Rab9 overexpression disrupts the actin cap formation and reduces the cortical actin levels. Mechanically, Rab9‐OE increases ROS levels, decreases mitochondrial membrane potential, ATP content and the mtDNA/nDNA ratio. Further studies showed that Rab9‐OE activates the PINK1‐PARKIN mitophagy pathway. Importantly, we found that reducing RAB9 protein expression in old oocytes could partially improve the rate of old oocyte maturation, ameliorate the accumulation of age‐related ROS levels and spindle abnormalities, and partially rescue ATP levels, mtDNA/nDNA ratio, and PINK1 and PARKIN expression. In conclusion, our results suggest that RAB9 is required to maintain the balance between mitochondrial function and meiosis, and that reducing RAB9 expression is a potential strategy to ameliorate age‐related deterioration of oocyte quality.

## Graphical abstract

This study showed that RAB9 protein accumulates in old human and mouse oocytes. RAB9 accumulation disrupted spindle formation, chromosome alignment and actin cap formation, increased ROS levels, decreased mitochondrial membrane potential, ATP content, mtDNA/nDNA ratio and activated the PINK1‐PARKIN pathway. Importantly, knockdown of RAB9 in old oocytes could partially improve the maturation rate of old oocytes and rescue mitochondrial function. These results suggest that RAB9 is required to maintain the balance between mitochondrial function and meiosis, and that reducing RAB9 expression is a potential strategy to ameliorate the age‐related decline in oocyte quality.

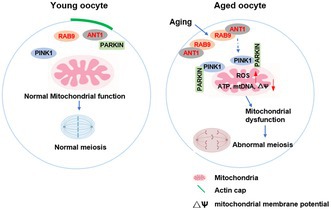

**Abbreviations**
ATP: adenosine 5'‐triphosphate
COCs: cumulus‐oocyte complexes
DIC: digital image correlation
GV: germinal vesicle
GVBD: germinal vesicle breakdown
hCG: human chorionic gonadotropin
IF: immunofluorescence
MI: Metaphase I
MII: Metaphase II
PB1: first polar body
PMSG: pregnant mare serum gonadotropin
Rab9‐KD: Rab9 knockdown
Rab9‐OE: Rab9 overexpression
ROS: reactive oxygen species
RT‐qPCR: real‐time qualitative polymerase chain reaction
WB: Western blotting

## 1 INTRODUCTION

The postponement of marriage and childbearing in women of reproductive age leads to an increased incidence of age‐related female infertility. Compared to young women, the IVF pregnancy rate in older infertile women is significantly reduced, and the likelihood of aneuploidy in MII oocytes is significantly increased (Mai et al., 2013). The accelerated decline in the number and quality of oocytes are the key factors affecting fertility in older women. Numerous studies have shown that the decline in oocyte quality in older women is associated with increased oocyte or embryo aneuploidy and genomic instability, possibly caused by epigenetic errors, or metabolic insults (Ivanova et al., 2017; Jones & Lane, 2013; Liu et al., 2023; Mikwar et al., 2020; Wu et al., 2022; M. Q. Zhang et al., 2020), the increased DNA damage and reactive oxygen species (ROS) (Rodríguez‐Nuevo et al., 2022; Wang et al., 2021), the disruption of spindle organization and mitochondrial function and membrane potential (May‐Panloup et al., 2016; Mihajlovic et al., 2023; Wasserzug‐Pash et al., 2022), premature centrosome separation, and deterioration of the cytoplasmic quality in oocytes (Liu et al., 2023; Mihajlovic et al., 2023). However, the molecular mechanism of oocyte aging is not fully understood. Recently, a large number of transcriptomes of GV and MII oocytes from women of different reproductive ages suggested that hundreds of genes are affected, especially in MII oocytes (Llonch et al., 2021; Reyes et al., 2017; Yuan et al., 2021). These results provide an important reference for the analysis of the molecular mechanism of oocyte aging and the search for therapeutic strategies.

RAB proteins are small GTPases that act as key regulators of intracellular vesicular trafficking. By cycling between GDP‐bound and GTP‐bound forms, RABs are coupled to recognition events in vesicle docking, function in membrane identity, vesicle formation and movement of vesicles, endocytosis, recycling, and degradation of proteins (Shan & Sun, 2021; Zerial & McBride, 2001). To data, more than 70 RAB and RAB‐like proteins have been identified (Burguete et al., 2008). Previous studies have shown that RAB proteins facilitate the movement of transport vesicles on the actin and microtubule cytoskeleton by co‐operating with actin and microtubule motor proteins and play an important role in oocyte meiosis and fertilization (Hou et al., 2016; Jin et al., 2022; Ma et al., 2014; Pan et al., 2019; Wang et al., 2019; Y. Zhang et al., 2019; Zou et al., 2021). For example, RAB35 mediates actin assembly and is required to facilitate spindle migration and meiotic maturation of mouse oocytes (Wang et al., 2016; Zhang et al., 2019). Deletion of RAB24 results in abnormal spindles, lagging chromosomes and failure of maturation, ultimately leading to aneuploidy in mouse oocytes (Qiu et al., 2019). RAB7, which accumulates at the spindle periphery and cortical region in oocytes to modulate mitochondrial function, and RAB7 activity is required to improve age‐related decline of oocyte quality (Jin et al., 2022; Pan et al., 2020). The RAB9 protein was mainly involved in cargo transport between early endosomes, late endosomes, and the trans‐Golgi network (Alfonzo‐Méndez et al., 2017; Kucera et al., 2016). It's been reported that RAB9 plays an important role in the PARKIN‐dependent mitophagy pathway, which is a critical to mitochondrial quality control mechanism (Jiang & Ogretmen, 2013). However, the role of RAB9 during oocyte meiosis and oocyte aging remains unknown.

In the present study, we found that the expression of RAB9 protein is upregulated in human and mouse old oocytes. Overexpression of Rab9 inhibits the extrusion of the first polar body (PB1) and reduces the developmental potential of oocytes. Further studies showed that Rab9 overexpression activates the PINK1‐PARKIN mediated mitophagy pathway, decreases mitochondrial function, increases ROS levels, and disrupts the actin filament assembly and spindle migration during oocyte meiosis. Importantly, knockdown of Rab9 in mouse old oocytes partially rescued the age‐related decline in oocyte quality.

## 2 MATERIALS AND METHODS

### 2.1 Ethics approval

This study was approved by the Institutional Review Board of Reproductive Medicine, Guangdong maternal and Child Health Hospital (project identification code: 202201059). All procedures performed in this study involving human participants were in accordance with the ethical standards of the Institutional Research Committee. Every human participant provided written informed consent.

### 2.2 Mice

Animal experimental protocols were approved by the Animal Care and Use Committee of Sun Yat‐sen University and were performed in accordance with institutional guidelines (project identification code: SYSU‐IACUC‐2024‐B1081). ICR mice were maintained on a 12/12 h dark–light cycle at 22°C with free access to food and water. Young oocytes were collected from 4 to 6 weeks old female ICR mice, and old oocytes were collected from approximately 12 months old reproductive aged female mice.

### 2.3 Antibodies

Primary and secondary antibodies were obtained from the following commercial sources: Mouse monoclonal anti‐α‐tubulin‐FITC antibody was purchased from Sigma (St Louis, MO, USA, T6074). Rabbit monoclonal anti‐RAB9 antibody was purchased from Abcam (Cambridge, MA, USA, EPR13272). Cy5‐conjugated goat anti‐human IgG and Cy5‐conjugated goat anti‐rabbit IgG were purchased from Jackson Immuno Research Laboratory (West Grove, PA, USA). Human anti‐centromere CREST antibody was purchased from Fitzgerald Industries International (Concord, MA, USA). OCT4 antibody (Santa Cruz Biotechnology), NANOG antibody (Abcam), PINK1 antibody (Abcam, ab137361), PARKIN antibody (Abcam, S378). In Situ Cell Death Detection Kit was purchased from Roche (Basel, Switzerland, 11,684,817,910). Other chemicals were purchased from Sigma Chemical Co. unless otherwise stated.

### 2.4 Oocyte collection and culture

Female ICR mice were euthanised after intraperitoneal injection of 10 IU pregnant mare serum gonadotropin (PMSG) for 46 h. Cumulus‐oocyte complexes (COCs) were obtained by manual rupture of antral ovarian follicles. Cumulus cells were removed by repeated pipetting under M2 medium. Oocytes were cultured in M16 medium under liquid mineral oil at 37°C in a 5% CO_2_ atmosphere for in vitro maturation. The number of oocytes at different developmental stages was recorded at 3 h germinal vesicle breakdown (GVBD), 8 h metaphase I (MI) and 14 h metaphase II (MII) after in vitro culture. The PB1 extrusion rate at 14 h represented the oocyte maturation rate.

### 2.5 Zygote collection and culture

For zygote collection and culture, 6–8 weeks old female mice were injected with 10 IU human chorionic gonadotropin (hCG) after 46 h of 10 IU PMSG injection, and then mated with the male mice. Zygotes were collected from the oviducts, digested with hyaluronidase, and then cultured in KSOM medium at 37°C, 5% CO_2_. The number of embryos at different developmental stages was recorded. The cleavage rate at 24 h represented the percentage of 2‐cell embryos in relation to the cultured zygotes, and the cleavage rate at 96 h represented the percentage of blastocysts.

### 2.6 Plasmid construction and mRNA synthesis

The PCR products were purified, digested with NotI and BamHI (NEB Inc., MA, USA) and then cloned into the PCDNA‐eGFP+ vector with six GFP tags. On the other hand, eGFP‐Rab9 plasmids were digested with NheI and BamHI, and then used to synthesise *Rab9* mRNA without GFP tags. For the synthesis of *Rab9* mRNA, plasmids were linearized with XBaI, mRNAs were produced and tailed by in vitro transcription using T7 mMESSAGE mMACHINE (Ambion, CA, USA) according to the manufacturer's instructions. Synthesized RNA was aliquoted and stored at −80°C.

### 2.7 Microinjection of siRNA and mRNA

GV oocytes and zygotes were microinjected with Rab9 siRNA or mRNA using an Eppendorf microinjector under an inverted microscope. For overexpression experiments, approximately 10 pL of *Rab9* mRNA solution (10 ng/μL) was injected into the cytoplasm of GV oocytes. In the control group, the same amount of RNase‐free PBS was injected. For knockdown experiments, the designed Rab9 siRNA was diluted to 20 μM with RNase‐free water, and approximately 10 pL of siRNA solution was injected into GV oocytes. The negative control was injected with the same amount of RNase‐free water. After siRNA injection, the oocytes were arrested at the germinal vesicle (GV) stage for 20 h to thoroughly deplete Rab9. The oocytes were then cultured in M16 medium at 37°C, 5% CO_2_ after washing.

### 2.8 Western blotting (WB)

Oocytes were lysed with RIPA lysate containing protease inhibitor. Protein loading buffer was then added and denatured at 100°C water for 5 min. Oocytes in each group were loaded and separated by 15% SDS‐PAGE gel. After electronic transfer, the PVDF membranes were blocked in 5% low‐fat dry milk for at least 1 h and then incubated overnight at 4°C with specific antibodies (rabbit anti‐RAB9 antibody, 1:1000; mouse anti‐ACTIN antibody 1:3000–5000). The membranes were then washed three times with TBST and incubated with secondary antibodies for 1 h at room temperature. After washing with TBST, the protein bands were visualised using an ECL and Western blotting detection system (GE Healthcare, Piscataway, NJ, USA).

### 2.9 Immunofluorescence (IF)

Oocytes or embryos were fixed with 4% paraformaldehyde (PFA) for 30 min and permeabilized with 0.5% Triton X‐100 for 20 min at room temperature. After three washes with PBS, the samples were blocked with 1% BSA for 1 h and then incubated with primary antibody overnight at 4°C. After washing, the samples were incubated with secondary antibody for 1 h at room temperature. DNA was counterstained with DAPI for 15 min. Finally, the oocytes were mounted on glass slides and imaged using a confocal microscope (Zeiss, SP8).

For F‐actin staining, MII oocytes were fixed in 3.7% PFA for 5 min, and blocked in 1% BSA for 1 h. Samples were incubated with FITC‐conjugated phalloidin for 1 h. For mitochondrial staining, oocytes were cultured in M2 medium containing 200 nM Mito Tracker Red (Molecular Probes, Eugene, OR) for 30 min at 37°C. To evaluate mitochondrial membrane potential, GV oocytes were cultured in M16 medium containing 2 μΜ JC‐1 (Biyuntian, C2006) for 30 min at 37°C. Chromosomes were counterstained with propidium iodide (PI) or Hoechst33342 for 10 min. After washing, the oocytes were transferred to a live cell imaging dish. Fluorescence was detected and imaged using a Leica SP8 inverted microscope. To measure ROS levels, MII oocytes were incubated in M2 medium with 5 μΜ CM‐H2DCFDA (Life Technologies, Invitrogen TM, C6827) for 30 min at 37°C in a 5% CO_2_ atmosphere. After washing, oocytes were transferred to a live cell imaging dish and observed using a confocal microscope.

### 2.10 Quantitation of ATP

The adenosine 5′‐triphosphate (ATP) bioluminescent somatic cell assay kit (Sigma, FLASC) was used to detect the ATP content of oocytes according to the manufacturer's protocol. Briefly, individual oocytes in each group were washed three times with PBS and then transferred to 100 μL of ATP assay mix working solution for 3 min at room temperature. To a separate vial containing 100 μL of 1× somatic cell ATP releasing reagent, add 50 μL ultrapure water and 50 μL oocyte lysis solution. Shake and transfer 100 μL to the reaction vial in an opaque 96‐well plate, and immediately measure the amount of light emitted [L_(SAM)_] using a luminometer. Add 50 μL ATP standard solution (500 fmol) and 50 μL oocyte lysis solution to a separate vial containing 100 μL 1× somatic cell ATP release reagent. Swirl briskly, transfer 100 μL to the reaction vial, and immediately measure the amount of light emitted [L_(SAM + IS)_] using a luminometer (GloMax 96 Microplate Luminometer, Promega, USA). The amount of ATP per oocyte can be calculated using the following equation: ATP = 500 fmol × L_(SAM)_/[L_(SAM + IS)−_L_(SAM)_].

### 2.11 Analysis of mtDNA/nDNA ratio

Relative mitochondrial DNA/nuclear DNA (mtDNA/nDNA) was assessed in mice as previously described. Briefly, genomic DNA was isolated from 10 oocytes in each group using the quick extract™ DNA extraction solution 1.0 (Lucigen, QE09050), according to the manufacturer's protocol: 5 μL of extraction buffer was added to the tube and incubated at 65°C for 3 h and at 95°C for 10 min. RT‐qPCR was then performed in 10 μL reactions using a Light Cycler 480II system (Roche Diagnostics). ND1 primer (forward: 5′‐CTAGCAGAAACAAACCGGGC‐3′, reverse: 5′‐CCGGCTGCGTATTCTACGTT‐3′) was used to detect mtDNA and HK2 primer (forward: 5′‐CTAGCAGAAACAAACCGGGC‐3′, reverse: 5′‐GGGAACACAAAAGACCTCTTCTGG‐3′) was used to detect nDNA. The analysis of the mtDNA/nDNA ratio can be calculated according to the classical ΔΔCt method used for qPCR analysis. First, ΔCt is calculated using the following formula: ΔCt = Ct (mtDNA gene)‐Ct (nDNA gene). Then, calculate ΔΔCt using the following formula: ΔΔCt = ΔCt (sample of interest)‐ΔCt (control sample). Finally, calculate the expression of each sample as 2^−ΔΔCt^ and import the data into GraphPad Prism for statistical analysis.

### 2.12 Statistical analysis

Data are presented as mean ± SEM, unless otherwise indicated. Statistical comparisons were made using Student's *t*‐test and ANOVA where appropriate. *p* < 0.05 was considered to be significant.

## 3 RESULTS

### 3.1 The accumulation of RAB9 is a barrier to meiosis in human oocytes

**FIGURE 1:**
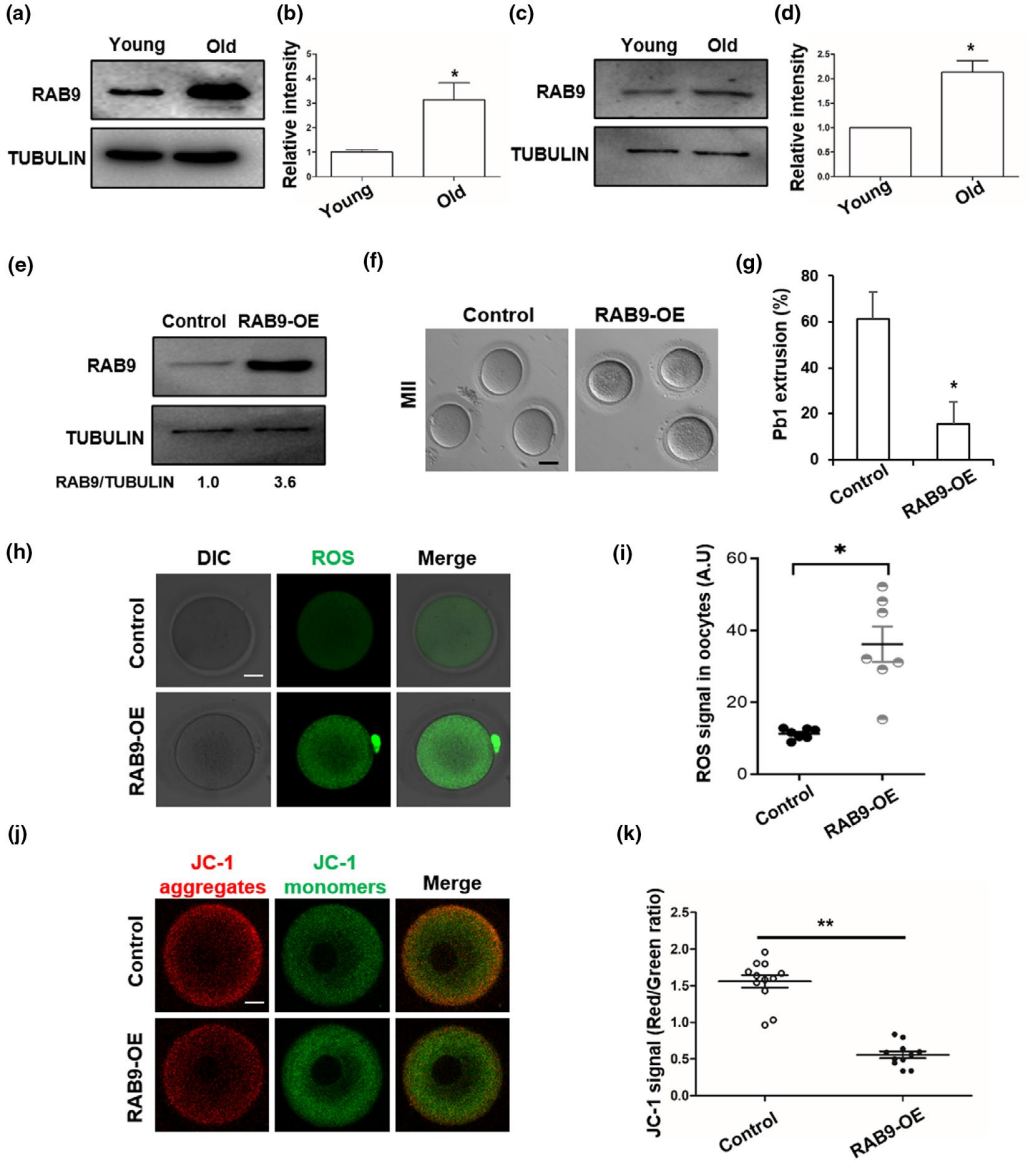
Accumulation of RAB9 impair human oocyte maturation. (a) RAB9 expression in young (age <35) and old (age >35) human granulosa cells. (b) Relative expression of RAB9 in young and old granulosa cells. *p < 0.05. (c) RAB9 expression in young (age <35) and old (age >35) human GV oocytes. (d) Relative expression of RAB9 in young and old GV oocytes. *p < 0.05. (e) Overexpression of RAB9 (RAB9‐OE) protein after mRNA injection was confirmed by WB. (f) Representative images of oocyte maturation in control and RAB9‐OE oocytes. Scale bar: 100 μm. (g) First polar body (Pb1) extrusion rate in control (*n* = 26) and RAB9‐OE (*n* = 28) oocytes. *p < 0.05. (h) Representative ROS images in control and RAB9‐OE oocytes. Scare bar: 25 μm. (i) ROS levels in control and RAB9‐OE oocytes. *p < 0.05. (j) Representative images of mitochondrial membrane potential in control and RAB9‐OE oocytes. Scale bar: 25 μm. (k) The JC‐1 aggregates (red)/monomers (green) fluorescence ratio in the control (*n* = 12) and RAB9‐OE (*n* = 11) oocytes. **p < 0.01.

By analysing the published proteomics of young and old oocytes, we found that the RAB9 protein is upregulated in old oocytes (Schwarzer et al., 2014). To further investigate whether the RAB9 protein is increased in old oocytes, we collected discarded granulosa cells and GV stage oocytes from female patients for WB, and divided the granulosa cells and oocytes into young (<35 years old) and old groups (≥35 years old) according to the age of the female. The results showed that the expression of RAB9 protein was significantly increased in granulosa cells and GV oocytes of the old group (Figure 1a–d). To explore the role of increased RAB9 protein in old oocyte meiosis, we upregulated the expression of RAB9 in young GV oocyte by microinjection of *RAB9* mRNA. WB result showed that the expression of RAB9 could be successfully increased after injection with *RAB9* mRNA (Figure 1e), and RAB9‐OE significantly decreased the rates of PB1 extrusion (Figure 1f,g). In addition, we found that RAB9‐OE significantly increased the ROS levels compared to control oocytes (Figure 1h,i). The mitochondrial membrane potential was then assessed by JC‐1 staining, and the result showd that the JC‐1 aggregates/monomers ratio was significantly reduced in RAB9‐OE oocytes (Figure 1j,k).

### 3.2 Increased RAB9 inhibits meiosis in mouse young oocytes

**FIGURE 2:**
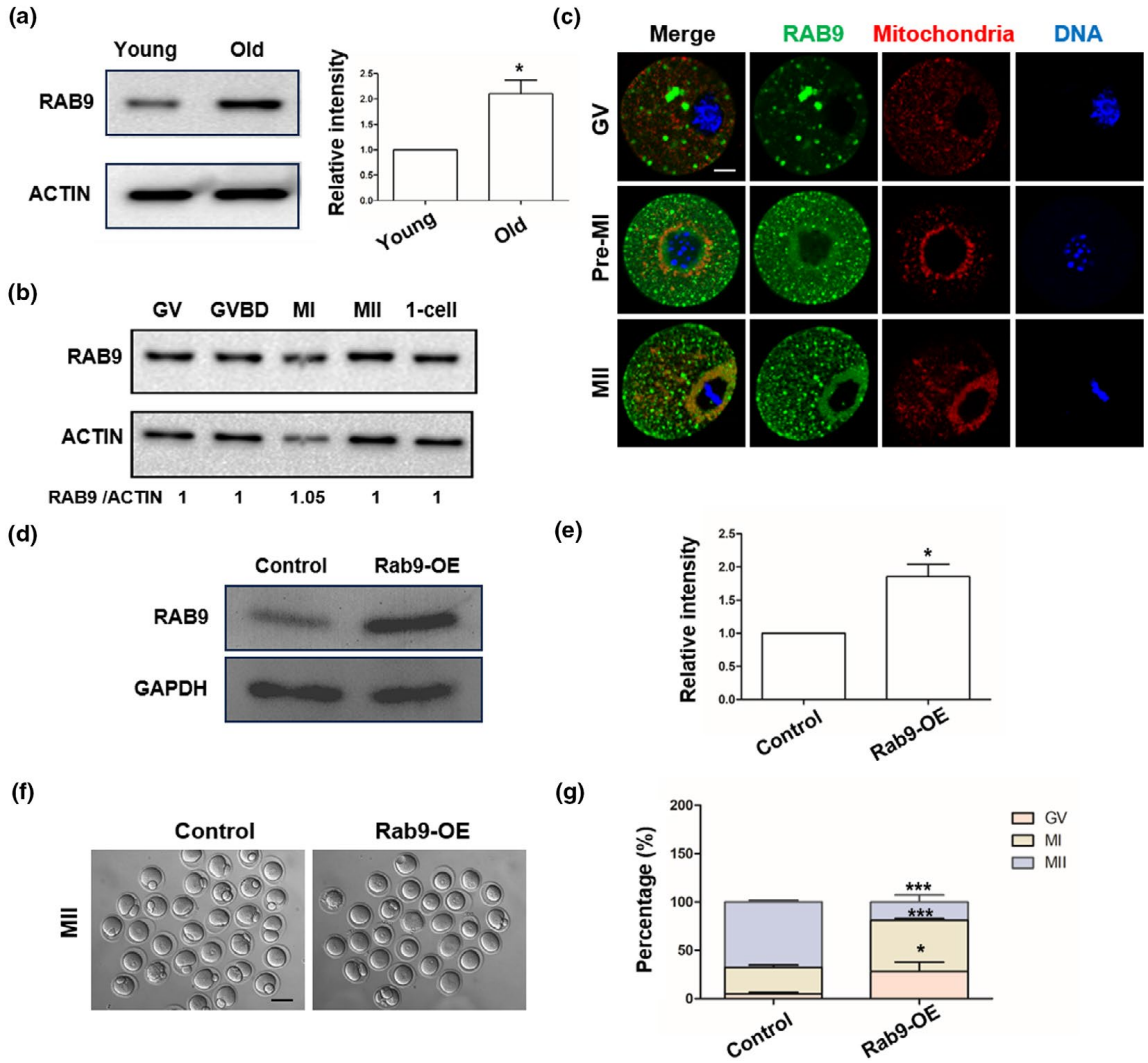
Effects of Rab9‐OE on mice oocyte maturation. (a) Relative RAB9 protein expression in young (4 weeks) and old (12 months) mouse oocytes. *p < 0.05. (b) RAB9 expression at GV (0 h), GVBD (3 h), MI (8 h), MII (14 h), and zygote stages. (c) Co‐immunostaining of RAB9 and mitochondria during mouse oocyte maturation. Green, RAB9; red, mitochondria; blue, DNA. Scale bar: 25 μm. (d) Western blots of RAB9 protein levels in control and Rab9‐OE oocytes. (e) Relative intensity of RAB9 protein in control and Rab9‐OE groups. GAPDH expression was used as an internal control. *p < 0.05. The band intensity was calculated using ImageJ software, and the ratio of RAB9/GAPDH expression was normalized. (f) Representative images of oocyte maturation in control and Rab9‐OE groups. Scale bar: 100 μm. (g) Quantitative analysis of GV, MI, and MII rates in control (*n* = 120) and Rab9‐OE (*n* = 112) groups. *p < 0.05, ***p < 0.001.

Due to the scarcity of human oocytes, we used mouse models to further investigate the function and potential mechanism of RAB9 in oocyte meiosis. Firstly, we collected GV oocytes from mice at different ages (4–6 week, 12 month) to determine the dynamics of RAB9. WB results showed that RAB9 protein was significantly increased in old oocytes compared to young oocytes (Figure 2a). Secondly, we examined the expression and localization of RAB9 protein in oocytes at different stages. WB indicated that the RAB9 expression levels were stable from the germinal vesicle (GV) stage to the zygote stage (Figure 2b). Since the mitochondrial membrane potential was decreased in RAB9‐OE oocytes (Figure 1j,k), we then examined the co‐localization of RAB9 with mitochondria using immunofluorescence staining. Our results showed that endogenous RAB9 was expressed as puncta and partially co‐localization with mitochondria (Figure 2c). In addition, we also examined the co‐localization of RAB9‐GFP with TUBULIN using IF, the result showd that RAB9‐GFP were without any overlap with TUBULIN (Figure S1). To investigate the role of RAB9 during meiotic maturation of mice oocytes, we knockdown or overexpressed Rab9 by microinjection of *Rab9* siRNA and mRNA, and the expression of RAB9 was evaluated by WB in the control and Rab9 knockdown or overexpression oocytes (Figure 2d,e and Figure S2A). The results showed that *Rab9* knockdown (*Rab9*‐KD) could successfully decrease the RAB9 protein level and did not affect oocyte maturation in mice (Figure S2A–C). The WB results showed that RAB9 protein could be significantly increased in RAB9 overexpression (RAB9‐OE) group (Figure 2d,e), and Rab9‐OE inhibited oocyte maturation and caused oocyte arrest at GV and MI stages, (Figure 2f,g).

### 3.3 Increased RAB9 is detrimental to the developmental potential of young oocytes

**FIGURE 3:**
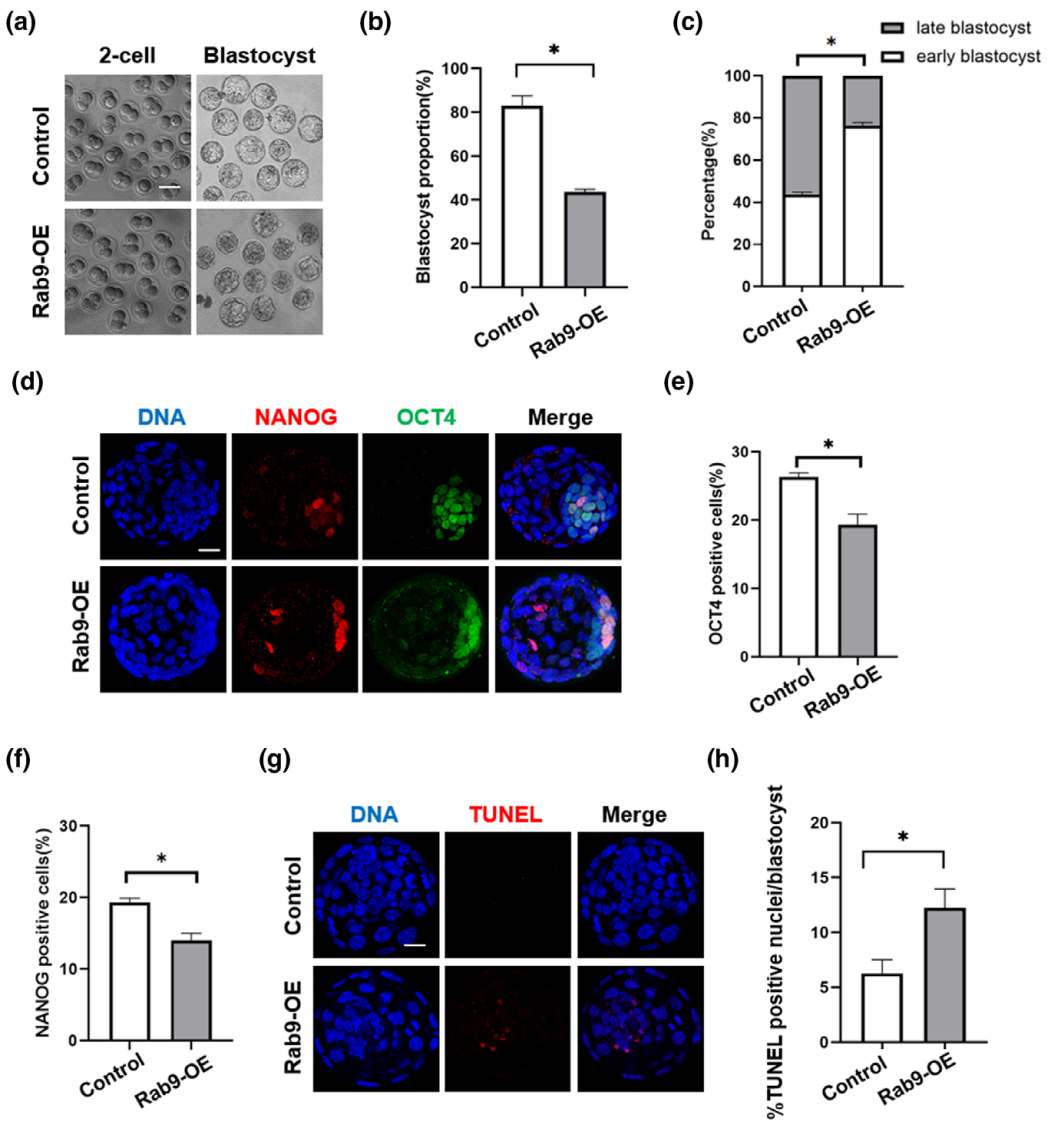
Rab9‐OE resulted in reduced oocyte developmental potential. (a) Representative images of 2‐cell and blastocyst stage embryos in control and Rab9‐OE oocytes. Scale bar: 100 μm. (b) Proportion of blastocysts in the control (*n* = 80) and Rab9‐OE (*n* = 80) groups. *p < 0.05. (c) The proportion of early and late blastocysts was statistically analyzed in the control (*n* = 90) and Rab9‐OE (*n* = 83) groups. *p < 0.05. (d) Representative images of late blastocysts labelled with OCT4 and NANOG in control and Rab9‐OE groups. Scale bar: 25 μm. (e, f) Quantification of the percentage of OCT4 and NANOG positive cells in late blastocysts. Control, Rab9‐OE: *N* = 89, 80. *p < 0.05. (g) Representative images of late blastocysts labelled with TUNEL in control and Rab9‐OE groups. Scale bar: 25 μm. (h) Statistical analysis of apoptotic signals per blastocyst in the control (*n* = 89) and Rab9‐OE (*n* = 68) groups. *p < 0.05.

To further investigate the role of RAB9 in oocyte developmental potential, we microinjected *Rab9* mRNA into the cytoplasm of MII oocytes and then examined the effect of Rab9‐OE on embryonic development after in vitro fertilization. The results showed that Rab9‐OE didn't affect cleavage, but significantly reduced the blastocyst rate (Figure 3a,b). We defined 32–64 cells as early blastocysts and more than 64 cells as late blastocysts and found that the proportion of late blastocysts was significantly reduced in the Rab9‐OE group (Figure 3c). The first cell lineage differentiation of embryos produced two cell populations: the trophectoderm and the inner cell mass, and the inner cell mass eventually differentiated into the fetus. The number and quality of the inner cell mass could reflect the embryonic developmental potential, OCT4 and NANOG are the specific markers of the inner cell mass and are essential for embryo development, so we used OCT4 and NANOG antibodies for immunofluorescence labelling of blastocysts. The results showed that the number of OCT4 positive cells and NANOG positive cells were significantly reduced in Rab9‐OE blastocyst compared to control blastocysts (Figure 3d–f). In addition, TUNEL staining showed an increased number of TUNEL positive cells among the blastocysts in the Rab9‐OE blastocyst (Figure 3g,h).

### 3.4 Rab9‐OE affects cytoskeletal organization during young oocyte maturation

**FIGURE 4:**
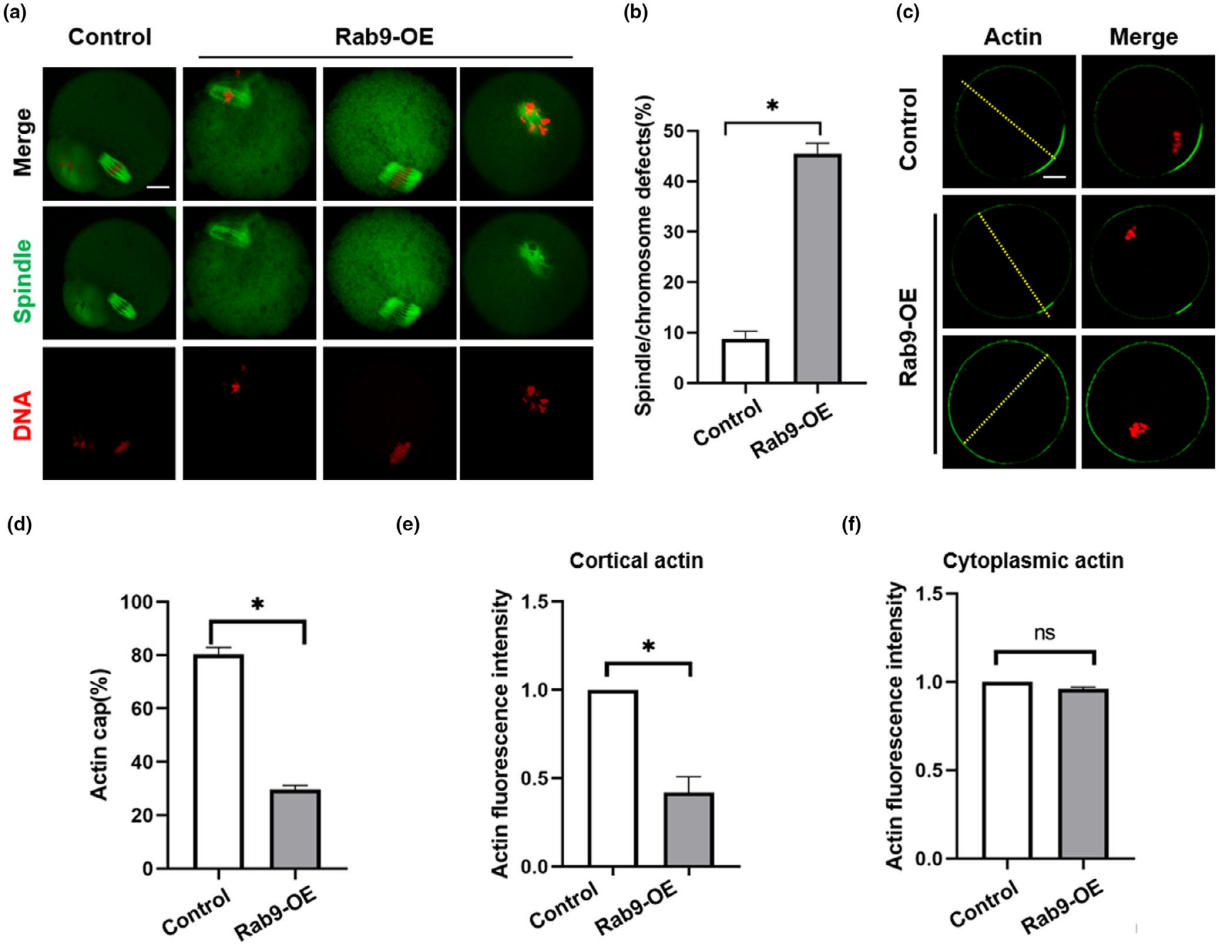
Effects of Rab9 overexpression on cytoskeletal organization. (a) Representative images of spindle morphology and chromosome alignment in control and Rab9‐OE oocytes. Scale bar: 25 μm. (b) Proportion of abnormal spindle/chromosome in the control (*n* = 100) and Rab9‐OE (*n* = 150) oocytes. *p < 0.05. (c) Representative images of Actin distribution in the control and Rab9‐OE oocytes. Scale bar: 25 μm. (d) The Actin cap ratio in control (*n* = 50) and Rab9‐OE (*n* = 40) oocytes. *p < 0.05. (e) The relative fluorescence intensity of cortical Actin in control and Rab9‐OE groups. *p < 0.05. (f) The relative fluorescence intensity of cytoplasmic Actin in control and Rab9‐OE groups.

Oocyte spindle assembly is essential for the PB1 extrusion. Previous studies have shown that other members of the RAB family play important roles in spindle formation (Shan & Sun, 2021), so we examined spindle morphology in the control and Rab9‐OE groups. The results showed that Rab9‐OE oocytes exhibited malformed spindles, multipolar spindles and the displacement of one or more chromosomes from the equator (Figure 4a). Statistical analysis confirmed that Rab9‐OE oocytes had a higher proportion of spindle or chromosome defects than the control group (Figure 4b). Since polar body extrusion is a cortical actin‐dependent process, we further examined the effect of Rab9‐OE on cortical actin distribution. We observed an increase in disrupted or absent actin caps with Rab9‐OE, and actin cap formation was significantly reduced in the Rab9‐OE group (Figure 4c,d). In addition, we found that Rab9‐OE didn't affect the cytoplasmic actin fluorescence intensity, but cortical actin fluorescence signals were significantly decreased at the cortex of the Rab9‐OE oocytes compared to control oocytes (Figure 4e,f).

### 3.5 Rab9‐OE inhibits mitochondrial function and increases mitophagy in mouse young oocyte meiosis

**FIGURE 5:**
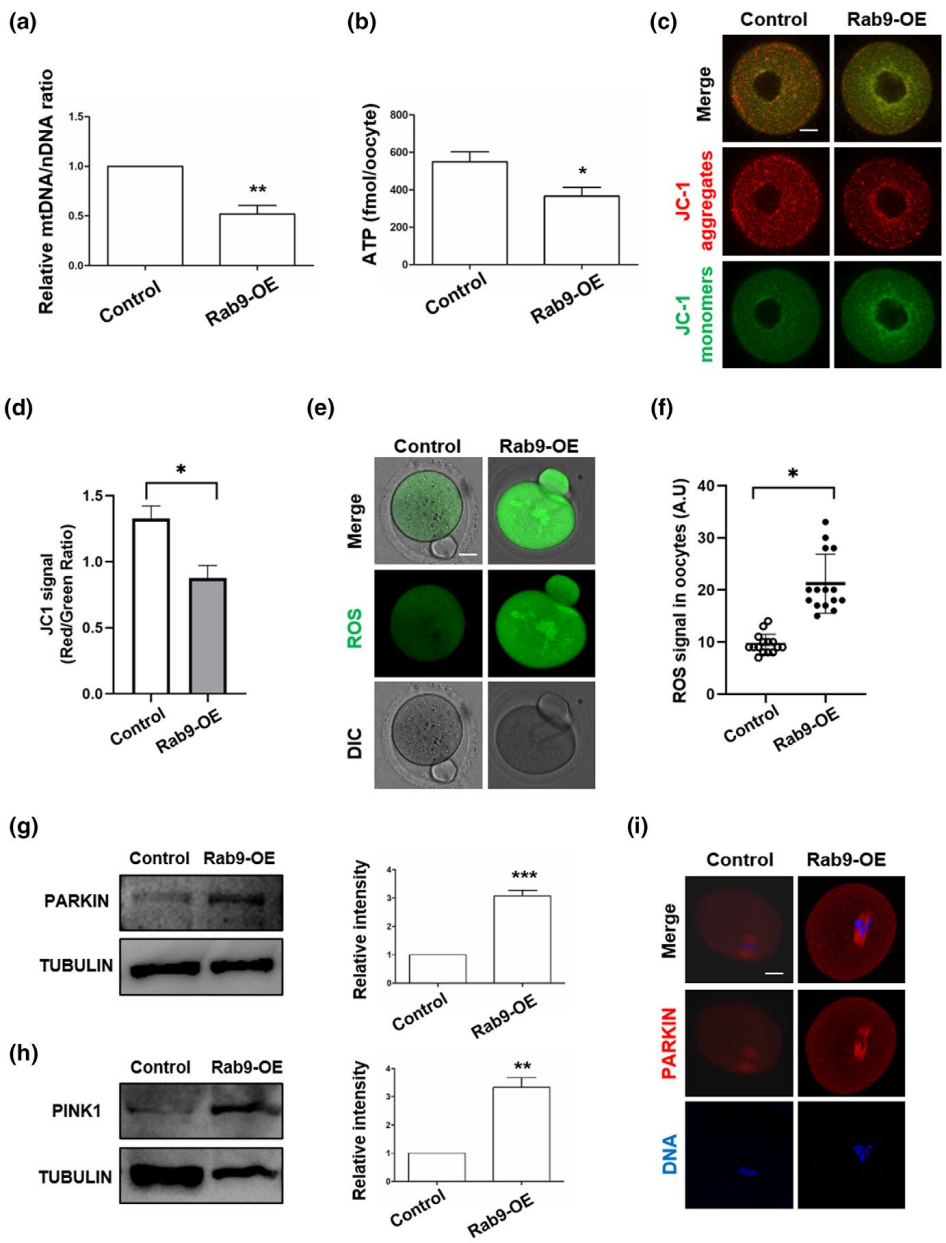
Rab9‐OE impairs mitochondrial function and activates the PINK1‐PARKIN signaling pathway in the mouse oocyte. (a) The relative mtDNA/nDNA ratio in control and Rab9‐OE groups. **p < 0.01.(b) The content of ATP per oocyte in control (*n* = 24) and Rab9‐OE (*n* = 24) groups. *p < 0.05. (c) Representative images of mitochondrial membrane potential in control and Rab9‐OE oocytes. Scale bar: 25 μm. (d) The JC‐1 red/green fluorescence ratio in control (*n* = 73) and Rab9‐OE (*n* = 75) group. Mitochondrial membrane potential (ΔΨm) was evaluated by JC‐1 staining as the ratio of red: Green signal densities. *p < 0.05. (e) Representative images of ROS in control and Rab9‐OE oocytes. Scale bar: 25 μm. (f) The ROS fluorescence intensity in control (*n* = 19) and Rab9‐OE (*n* = 15) groups. *p < 0.05. (g, h) Western blots of PINK1 and PARKIN protein levels in control and Rab9‐OE oocytes. **p < 0.01, ***p < 0.001. (i) Representative images of PARKIN in control and Rab9‐OE oocytes at MII stage. Scale bar: 25 μm.

Normal mitochondrial function is essential for maintaining oocyte development, and mitochondrial quality control plays an important role in maintaining mitochondrial homeostasis. It's been reported that Rab9 knockdown reduces mitophagy and maintains mitochondrial function in IGF‐IIR activated cardiomyocytes (Jiang & Ogretmen, 2013). We therefore investigated the role of RAB9 in the regulation of oocyte mitochondrial function. We found that the relative mtDNA/nDNA ratio and the amount of ATP were significantly reduced in Rab9‐OE oocytes (Figure 5a,b). Mitochondrial membrane potential is an important marker of mitochondrial function and we labelled oocytes with the fluorescent dye JC‐1 for mitochondrial membrane potential. The results showed that the JC‐1 red/green ratio was significantly decreased in Rab9‐OE oocytes (Figure 5c,d). Mitochondria are the major generators of ROS and one of the major targets of ROS‐induced oxidative damage, and the decrease in mitochondrial membrane potential is associated with increased ROS production. We assessed ROS in oocytes and showed that ROS levels were significantly increased in Rab9‐OE oocytes (Figure 5e,f).

The best‐known pathway of mitophagy involves the stabilisation of the mitochondrial kinase PINK1 on depolarised mitochondrial membranes, PINK1 recognises damaged mitochondria and recruits PARKIN to initiate mitochondrial autophagy. We found that Rab9‐OE significantly increased the expression of PINK1 and PARKIN (Figure 5g,h), and IF results also showed that Rab9‐OE enhanced PARKIN intensity (Figure 5i and Figure S3). To further explore the mechanism of RAB9 regulation of mitophagy, we performed IP‐MS in 293 T cells. We found that RAB9 could interact with ANT1, the mitophagy regulator (Figure S4).

### 3.6 Reducing Rab9 expression partially rescue maternal age‐related meiotic defects in mouse old oocytes

**FIGURE 6:**
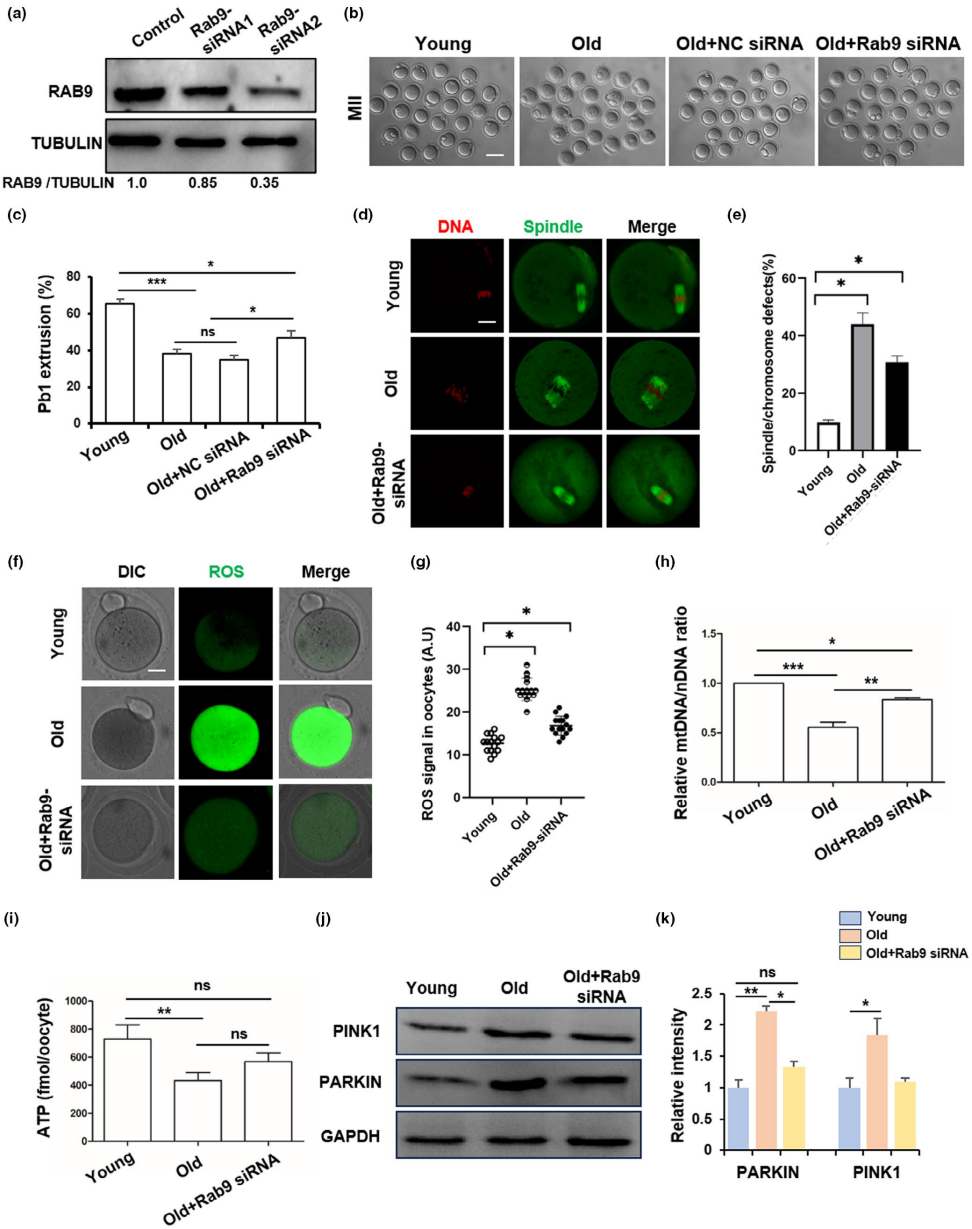
Rab9 knockdown partially ameliorated the oocyte quality of old mice. (a) Western blots of RAB9 protein levels in control and Rab9‐siRNA groups. (b) Representative images of oocyte maturation in young, old, old+NC siRNA and old+Rab9 siRNA groups. Scale bar: 100 μm. (c) First polar body (pb1) rates in young, old, old+NC siRNA and old+Rab9 siRNA groups. *p < 0.05, ***p < 0.001. (d) Representative images of spindles and chromosomes in young, old and old+Rab9 siRNA groups. Scale bar: 25 μm. (e) The rate of spindle and chromosome assembly abnormalities in control (*n* = 80), Old (*n* = 59) and Old+Rab9 siRNA (*n* = 78) groups. *p < 0.05. (f) Representative images of ROS in Young, Old, and Old+Rab9 siRNA groups. Scale bar: 25 μm. (g) The fluorescence intensity of ROS in young, old, and old+Rab9 siRNA groups. *p < 0.05. (h) The relative mtDNA/nDNA ratio in young, old, and old+Rab9 siRNA groups. *p < 0.05, **p < 0.01, ***p < 0.001. (i) ATP content per oocyte in young (*n* = 16), old (*n* = 16) and old+Rab9 siRNA (*n* = 15) groups. **p < 0.01. (j) Western blots of PARKIN in young, old, and old+Rab9 siRNA groups. (k) Relative intensity of PINK1 and PARKIN protein in young, old, and old+Rab9 siRNA groups. *p < 0.05, **p < 0.01.

Previous studies have shown that other members of the RAB family play important roles in oocyte aging (Jin et al., 2022; Shan & Sun, 2021). We found that the increased RAB9 protein is a barrier to oocyte meiosis in humans and mice, so we knockdown the expression of Rab9 by microinjection Rab9‐siRNA into old GV oocytes to investigate whether the quality of old oocytes could be improved. The WB result showed that the expression of RAB9 could be successfully decreased after microinjection of Rab9‐siRNA2 (Figure 6a), and Rab9‐siRNA2 was used for further study. The oocyte maturation experiment showed that the maturation rate was significantly decreased in old oocytes compared to young oocytes, and Rab9‐KD could partially rescue the maturation rate of old oocytes (Figure 6b,c). In addition, the rate of spindle defects was significantly increased in old oocytes compared to young oocytes, and Rab9‐KD could partially rescue the abnormal spindle in old oocytes (Figure 6d,e). We also found that ROS levels in old oocytes was significantly higher than that in young oocytes, and Rab9‐KD could significantly reduce the level of ROS in old oocytes (Figure 6f,g). Next, we investigated whether Rab9‐KD could rescue the mitochondrial function and mitophagy in old oocytes. The results showed that Rab9‐KD could partially improve the mtDNA/nDNA ratio and the amount of ATP in old oocytes (Figure 6h,i). In addition, Rab9‐KD could partially rescue the abnormal expression of PINK1 and PARKIN in old oocytes (Figure 6j,k, and Figure S5).

## 4 DISCUSSION

**FIGURE 7:**
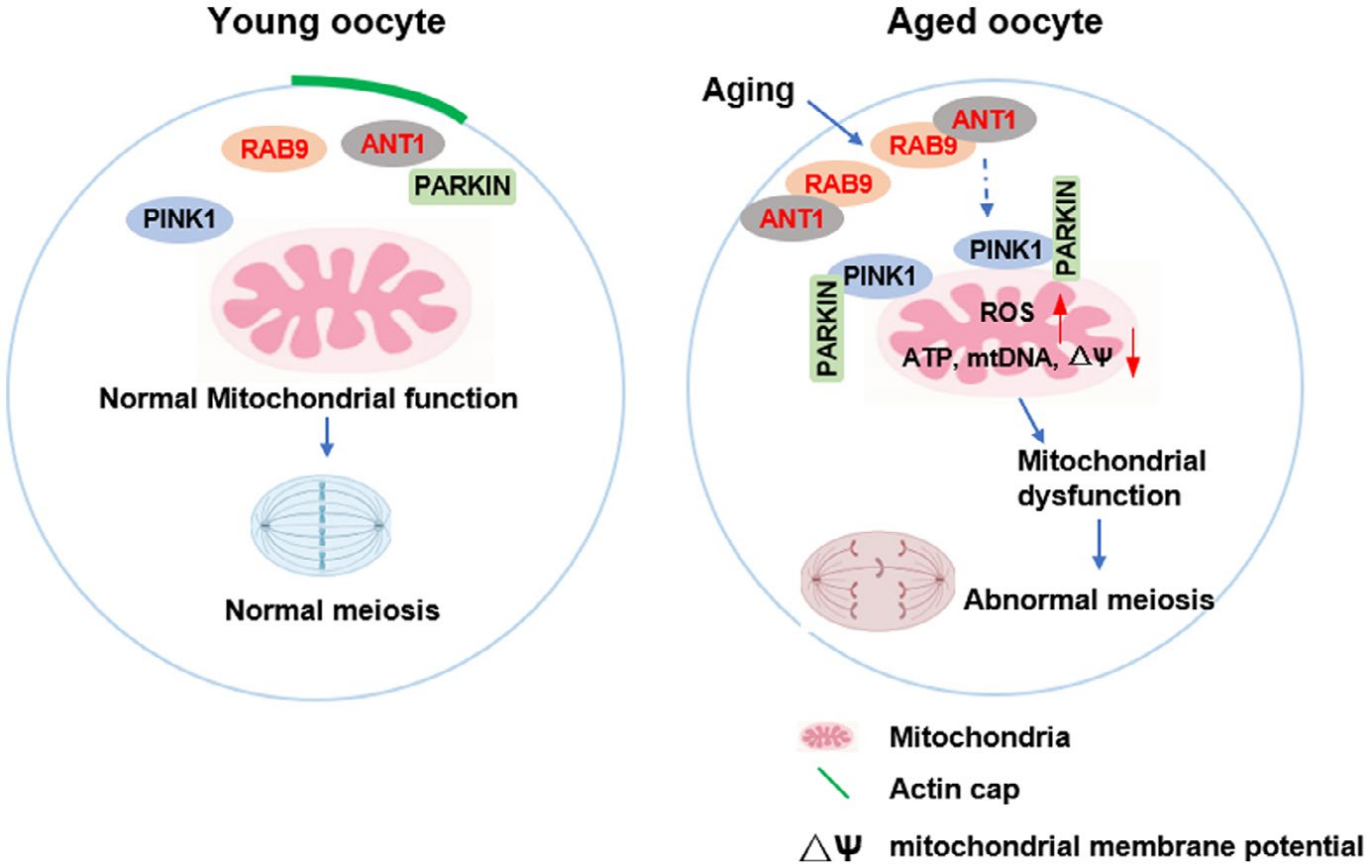
A schematic diagram depicting the role of RAB9 in oocyte meiosis and mitophagy during aging.

This study investigated the functions and potential mechanisms of RAB9 during meiotic maturation and aging of mouse oocyte. We showed that the expression of RAB9 protein was significantly increased in aged human and mouse oocytes. Further studies showed that the accumulation of RAB9 activated the PINK1‐PARKIN mediated mitophagy pathway and led to mitochondrial dysfunction, increased ROS levels and disrupted the cytoskeletal organization, which ultimately inhibited oocyte meiosis and induced oocyte aging (Figure 7).

RAB GTPase family number, including RAB5, RAB6A, RAB8A, RAB11, RAB23, RAB24, RAB35, have been reported to function in oocyte meiosis (Hou et al., 2016; Ma et al., 2014; Ma et al., 2016; Pan et al., 2019; Qiu et al., 2019; H. H. Wang et al., 2019; Y. Zhang et al., 2019). It's worth noting that RAB7 activity is required to maintain the balance between mitophagy and chromosome stability, and RAB7 activator is a good candidate for improving age‐related decline in oocyte quality (Jin et al., 2022). The small GTPase RAB9 is a member of the RAB family and is known to be a key regulator of the late endosomal/lysosomal network (Alfonzo‐Méndez et al., 2017; Jiang & Ogretmen, 2013; Kucera et al., 2016). However, the function of RAB9 in oocyte meiosis and aging is unclear. In this study, we found that RAB9 protein expression is significantly increased in aged human and mouse oocytes, and increased RAB9 inhibits oocyte meiosis and reduces oocyte developmental potential.

Previous studies have shown that the RAB GTPase members RAB7, RAB8A, RAB14, RAB35 are present in mammalian oocytes and localise to the oocyte cortex and accumulate at the periphery of the spindle (Jin et al., 2022; Pan et al., 2019; Y. Zhang et al., 2019; Zou et al., 2021). Here, we found that RAB9 is expressed at all the stages of meiosis. RAB9 localization at the oocyte cortex and accumulated at the periphery of the spindle. In addition, co‐staining results showed that RAB9 could partially co‐localization with mitochondria. Further studies showed that the rate of PB1 extrusion was reduced and oocytes were blocked at the GV and MI stages after Rab9 overexpression. Actin is known to be particularly important for microtubule migration and anchoring, which determines PB1 formation (Londoño‐Vásquez et al., 2022; Ye & Homer, 2022). Our previous study showed that cortical actin cap formation and cytoskeletal organization are essential for PB1 extrusion and oocyte aging in mice (Gao et al., 2022). Here, we found that the high incidence of spindle defects, chromosome disorganization and loss of actin cap in Rab9 overexpression oocytes. Similar findings have been reported for other RAB GTPases family members in oocytes (Shan & Sun, 2021). Previous studies have confirmed that mitochondrial dysfunction in oocytes is a possible mechanism involved in the poor developmental ability of oocytes in old women (Bentov & Casper, 2013; Ramalho‐Santos et al., 2009). For example, it's been reported that mitochondrial swelling, cristae alteration and abnormal mitochondrial morphology have been observed in patients aged >38 years (Lu et al., 2022). It has also been reported that increased ROS levels due to mitochondrial dysfunction can induce chromosome nondisjunction, spindle instability, telomere shortening, and reduced developmental competence in aged oocytes (Guo & Yu, 2019). A recent study showed that the small GTPase RAB7 is required to maintain the balance between mitophagy and chromosome stability, and that reducing RAB7 expression could ameliorate age‐related decline in oocyte quality (Jin et al., 2022). Our results showed that Rab9 overexpression increased the rate of abnormal mitochondria and ROS levels, and decreased the mitochondrial membrane potential. Taken together, these results suggest that RAB9 accumulation regulates mitochondrial homeostasis, affecting cytoskeletal structure and ROS levels, and ultimately regulating oocyte meiosis.

Adenine nucleotide translocase 1 (ANT1) is the most abundant protein in the inner mitochondrial membrane with ATP/ADP binding sites on its surface. Mice lacking ANT1 show delayed mitophagy, leading to a continuous accumulation of abnormal mitochondria (Hoshino et al., 2019). As a regulator of vesicle trafficking, we have shown that the RAB9 GTPase can interact with ANT1 and increase the expression of PINK1 and PARKIN. Activation of the classical PINK1/PARKIN mitophagy signaling pathway relies on the opening and closing of TIM23 membrane pore channels. When mitochondria are damaged or depolarized, TIM23 membrane pore channels close, blocking PARL's shearing of the key autophagy protein PINK1, resulting in the accumulation of PINK1 on the outer mitochondrial membrane, thereby recruiting PARKIN to promote the onset of mitochondrial autophagy. ANT1 directly binds to TIM44 of the TIM23 membrane pore channel complex and regulates TIM23 membrane pore channel closure, stabilizes PINK1 expression, and promotes the occurrence of mitophagy (Hoshino et al., 2019). Therefore, we hypothesized that RAB9 binds to ANT1 and recruits more ANT1 in aging oocytes. Under mitochondrial depolarization, ANT1 regulates the closure of TIM23 membrane pore channels and stabilizes the expression of PINK1, thereby recruiting PARKIN to promote mitophagy. Female fertility is one of the first bodily functions to be affected by aging, and the accelerated decline in oocyte number and quality is widely recognized as the major factor culminating in age‐related decline in fertility (Secomandi et al., 2022). Altered mitochondrial function, including reduced energy production, accumulation of mtDNA deletions/mutations or reduced oocyte mitochondrial membrane potential, has been associated with chromosomal nondisjunction during meiosis, and leads to reduced quality and developmental potential in aged oocytes and embryos (Lu et al., 2022; Nohales‐Córcoles et al., 2016). A previous study showed that RAB7 GTPase is required to maintain the balance between mitophagy and chromosome stability, and that reducing RAB7 expression could ameliorate age‐related decline in oocyte quality (Jin et al., 2022). As a regulator of the vesicle trafficking, RAB9 GTPase was significantly increased in human and mouse old oocytes, and reducing the accumulation of RAB9 protein in old mouse oocytes by knockdown Rab9 could partially rescue the old oocyte maturation, ameliorate the accumulation of age‐related ROS levels and spindle abnormalities. In addition, Rab9 knockdown could also partially rescue the mitochondrial dysfunction in old oocytes. Therefore, the accumulation of RAB9 protein is one of the factors that reduces the quality of old oocytes, and reducing the expression of Rab9 in old oocytes may be a potential strategy to improve oocyte quality in old women.

## 5 CONCLUSION

In this study, we found that the expression of RAB9 protein is significantly increased in old oocytes of humans and mice. The accumulation of RAB9 inhibits oocyte maturation and decreases oocyte developmental potential, Rab9‐OE activates the PINK1‐PARKIN pathway, disrupts the cytoskeletal organization and increases ROS levels, which ultimately inhibits oocyte meiosis and induces oocytes aging. Importantly, we found that reducing the accumulation of RAB9 protein in old oocytes could partially rescue the quality of old oocytes. We propose that inhibiting the expression of RAB9 may be a potential strategy in IVF clinics to improve oocyte quality in women of advanced maternal age.

## AUTHOR CONTRIBUTIONS

J. H. and F. L. conceived and designed the project. T. X. and M. G. drafted the manuscript. M. G. and F. W. performed the major experiments. T. X., H. L., T. C. and Y. Q. analyzed the data. S. L., W. W. and Y. Z. helped with animal housing and related experiments. J. H. and F. L. contributed to the final approval of the manuscript.

## FUNDING INFORMATION

This research was funded by the National Natural Science Foundation of China (Nos. 82201827 and 82271688), the Laboratory of Lingnan Modern Agriculture Project (NZ2021005), the Guangdong Basic and Applied Basic Research Foundation (2023A1515111005) and the Joint Program on Health Science & Technology Innovation of Hainan Province (WSJK2024QN027).

## CONFLICT OF INTEREST STATEMENT

The authors declare that they have no conflicts of interests.

## Supporting information


Data S1:

## Data Availability

All data generated during this study are included in this published article and its supplementary information files.
