# Supplementary material for: Age‐associated accumulation of RAB9 disrupts oocyte meiosis
Source: Aging Cell. 2024 Dec 15;24(4):e14449. doi: 10.1111/acel.14449 (PMC11984694; doi:10.1111/acel.14449)
Supplement: Supplementary file 1 — Data S1: [file ACEL-24-e14449-s001.pdf]

Supplemental-Figure 1

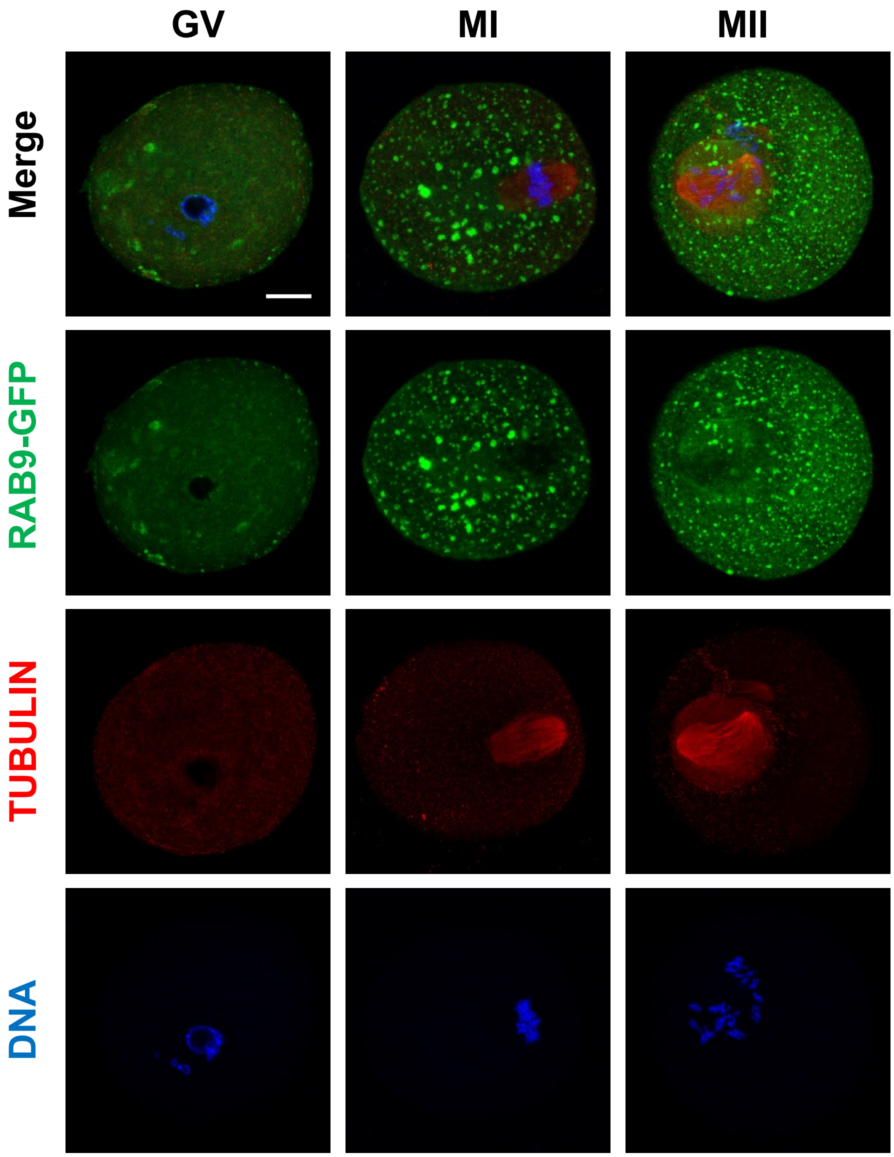

Figure S1. The co-staining of RAB9-GFP and TUBULIN in mice oocytes at different stages. Scale bar: 25 μm.

Supplemental-Figure 2

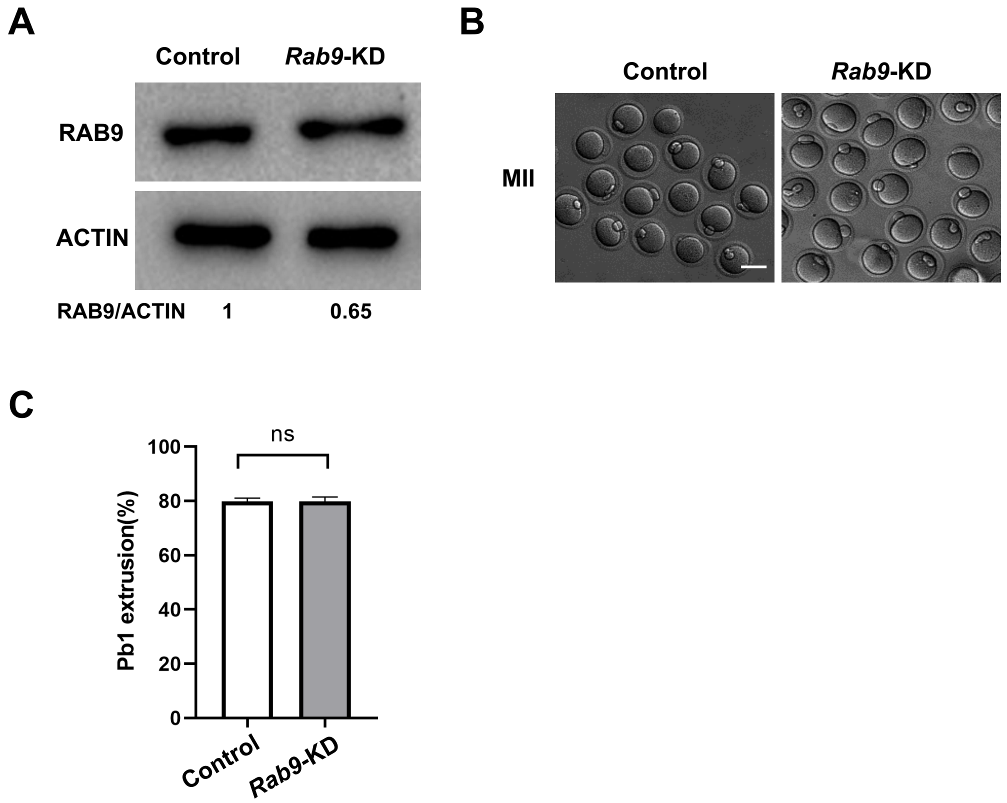

Figure S2. Effects of Rab9 knockdown on mouse oocyte meiosis. (A) Knockdown of endogenous Rab9 (Rab9-KD) protein after siRNA injection was confirmed by WB. (B) Representative images of control and Rab9-KD oocytes. Scale bar: 100 μm. (C) Quantitative analysis of Pb1 extrusion rate in control and Rab9-KD oocytes.

Supplemental-Figure 3

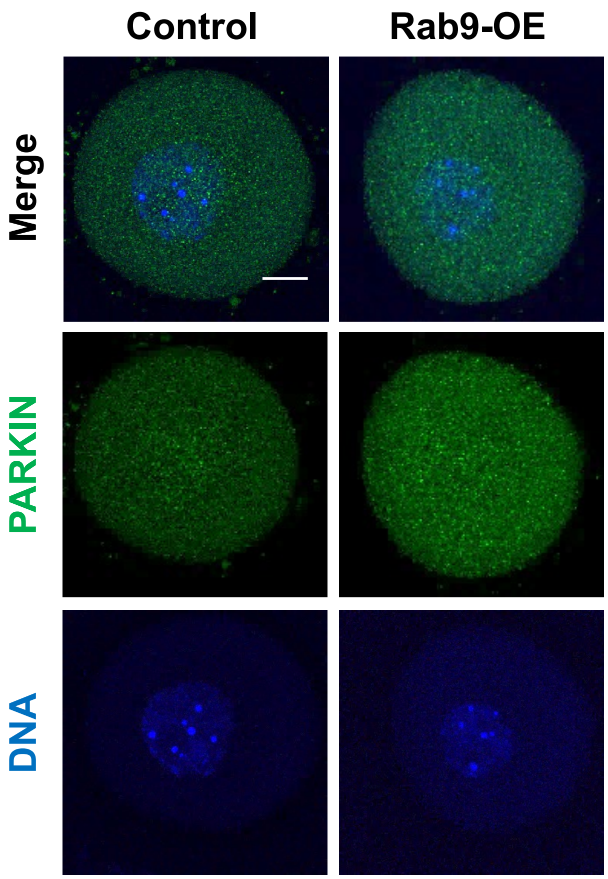

Figure S3. Representative images of PARKIN in control and Rab9-OE groups at GV stage. Scale bar: 25 μm.

Supplemental-Figure 4

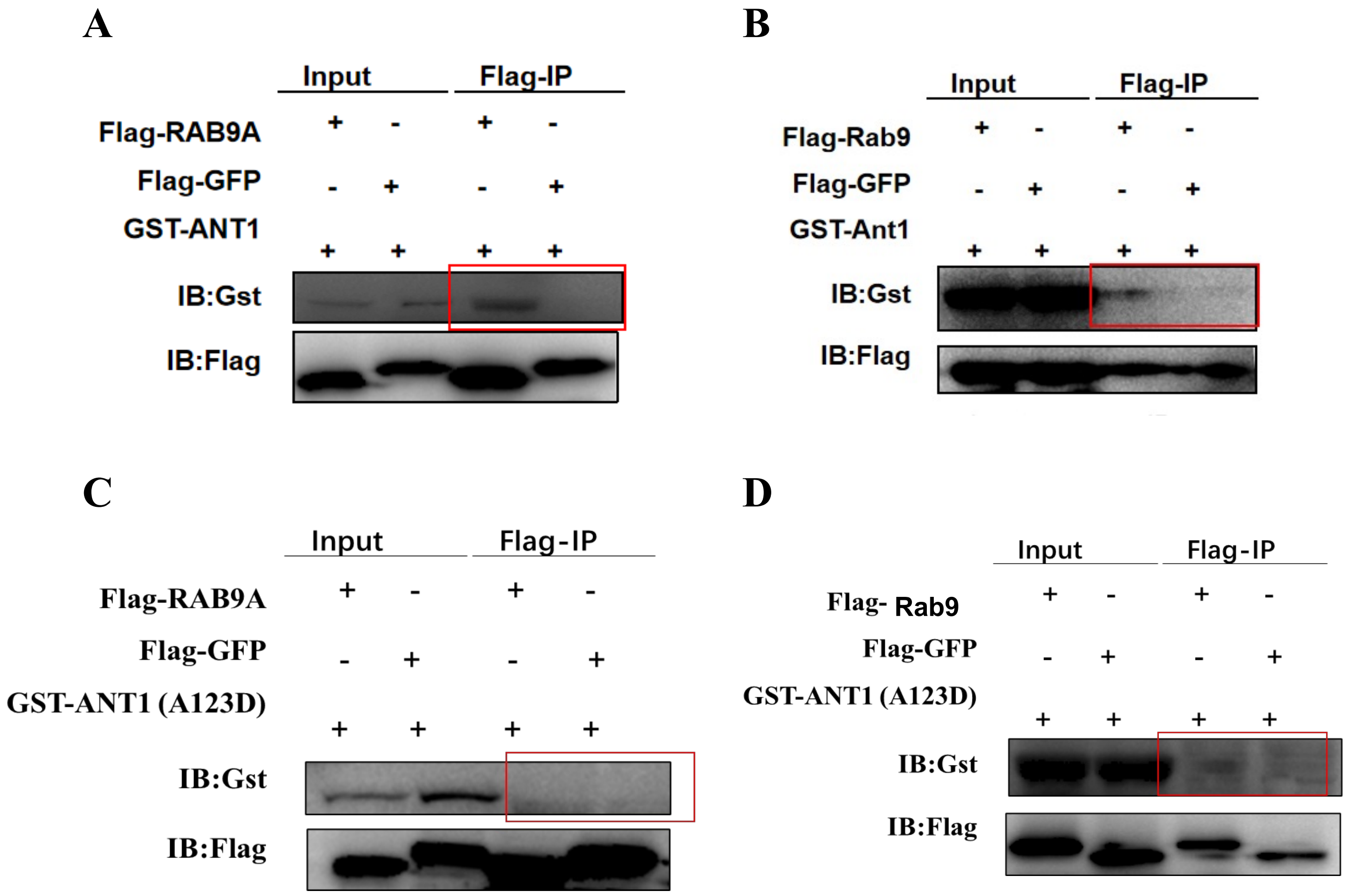

Figure S4. CO-IP between RAB9 and ANT1. (A) Human Flag-RAB9A and GST- ANT1 were co-transferred into 293T cells, and the cells were collected 48 hours later. After NETN lysis, the immunoprecipitation test was performed. Input and IP samples were tested by Western Blot. (B) Mice Flag-Rab9 and GST-Ant1 were transferred into 293T cells, and the cells were collected for Co-IP test 48 hours later. (C) Human Flag-RAB9A and GST- ANT1 mutant (A123D) were transferred into 293T cells, and the cells were collected for Co-IP test 48 hours later. (D) Mice Flag-Rab9 and GST-Ant1 mutant (A123D) were transferred into 293T cells, and the cells were collected for Co-IP test 48 hours later.

Supplemental-Figure 5

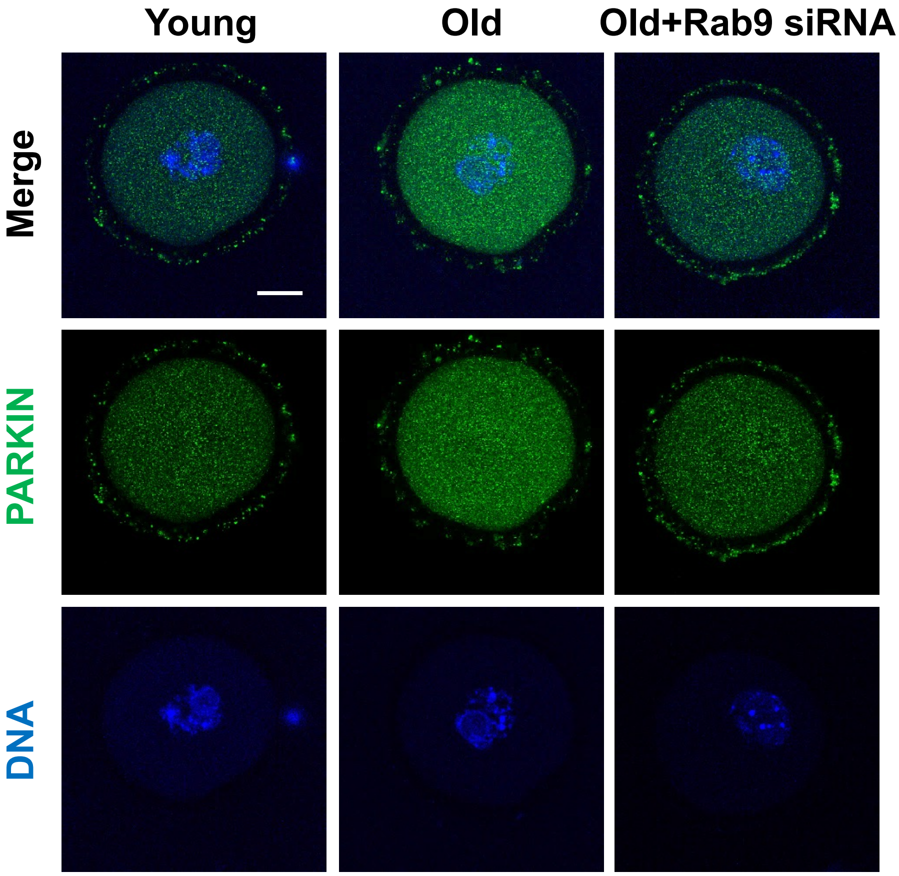

Figure S5. Representative images of PARKIN in young, old and old+Rab9 siRNA groups at GV stage. Scale bar: 25 μm.
